# Case Report: *Talaromyces marneffei* infections associated with pharyngeal and laryngeal injuries in three children with aberrant innate immunity: a case series

**DOI:** 10.3389/fped.2025.1615943

**Published:** 2025-10-09

**Authors:** Changhao Zhang, Gen Lu, Xuehua Xu, Huifeng Fan

**Affiliations:** Department of Respiration, Guangzhou Women and Children’s Medical Centre, Guangzhou Medical University, Guangzhou, Guangdong, China

**Keywords:** *Talaromyces marneffei*, children, pharyngeal, laryngeal, infection

## Abstract

**Background:**

*Talaromyces marneffei* (*T. marneffei*) is a pathogenic yeast that causes high fatality rates among immunocompromised individuals. The organism appears to target the lungs, liver, gut-associated lymphoid tissues, lymph nodes, spleen, bone marrow, kidneys, and the tonsils in the affected individuals. Evidence suggests that the lungs and liver are seriously injured in *T. marneffei* infections. Injuries to the pharyngeal and laryngeal compartments have seldom been reported.

**Case presentation:**

Three pediatric patients with *T. marneffei* infections were admitted to the Guangzhou Women and Children's Center between August 2015 and March 2023. All three children showed evidence of pharyngeal and laryngeal injuries.

**Conclusions:**

Damage to the pharyngeal and laryngeal compartments appears to be an important clinical manifestation among HIV-negative pediatric individuals with a *T. marneffei* infection.

## 1 Introduction

*Talaromyces marneffei* (*T. marneffei*) is an infectious agent with a high fatality rate. It was previously known as *Penicillium marneffei* and is a dimorphic fungus. It was first isolated at the Pasteur Institute in 1956 from the hepatic lesions of a Chinese bamboo rat that died of mycosis (1). *T. marneffei* often invades patients with compromised immune systems, especially human immunodeficiency virus (HIV)-positive children (2). In HIV-negative pediatric patients without additional secondary immunodeficiencies who are either born or reside in South China or other endemic regions, infections caused by *T. marneffei* may serve as a clinical indicator of underlying inborn errors of immunity (IEIs) (3). The immunology and genetics of these children need to be evaluated (4). *T. marneffei* infections result in systemic injury and a high mortality rate (5). The organism appears to target the lungs, liver, gut-associated lymphoid tissues, lymph nodes, spleen, bone marrow, kidneys, and the tonsils of affected individuals (6). Evidence suggests that the lungs and liver are severely injured by *T. marneffei* infections, whereas injuries to the pharyngeal and laryngeal compartments have rarely been reported. This retrospective study on three HIV-negative children infected with *T. marneffei* involved assessments of pharyngeal and laryngeal injuries, providing novel insights into the disease.

## 2 Case reports

Three pediatric patients with *T. marneffei* infections were admitted to the Guangzhou Women and Children's Center between August 2015 and March 2023. This study was approved by the ethics committee of Guangzhou Women and Children's Medical Center. Written informed consent was obtained from the parents.

### 2.1 Patient 1

In August 2015, a 13-year-old boy (P1) was hospitalized with hoarseness and dyspnea for more than 20 days. He was diagnosed with hyperimmunoglobulin E syndrome (HIES) and a *T. marneffei* infection. Nasopharyngolaryngoscopy revealed lesions affecting the pharyngeal and laryngeal regions.

### 2.2 Patient 2

In August 2019, a 1-year-old boy (P2) was hospitalized because of intermittent laryngeal stridor accompanied by a cough for more than 2 months, aggravated by hoarseness for 3 weeks. He was diagnosed with subglottic stenosis. Moreover, bronchoscopy showed injury to the glottic region.

### 2.3 Patient 3

In November 2021, a 1-year-old girl (P3) presented with pharyngeal ulcers, a fever, and a cough for 10 days. The patient was diagnosed with primary immunodeficiency disease and initially presented with ulcerative pharyngitis, which subsequently progressed to nasopharyngeal adhesion.

## 3 Results

### 3.1 Clinical findings

The clinical characteristics are summarized in Table 1. The laboratory results from the three patients with *T. marneffei* infections at the time of admission are summarized in Table 2. All three patients (P1–P3) were negative for HIV. Enzyme-linked immunosorbent assay kits were used to qualitatively detect HIV-1 and/or HIV-2 antibodies and P24 antigen in human serum or plasma samples. The serum immunoglobulin G (IgG) concentrations of the three patients were 17.1,12.9 and 42.4 g/L, respectively, and were slightly higher than the normal range. The serum immunoglobulin M (IgM) concentration was higher than the normal range in two patients (2.62 g/L in P2 and 1.86 g/L in P3). The serum immunoglobulin E (IgE) concentrations in two patients were also higher than the normal range (5,310 IU/mL in P1 and 422 IU/mL in P2). The lymphocyte counts in P1, including those of CD3 + CD4+ T cells (323.88 cells/µL), CD16 + CD56+ NK cells (75.91 cells/µL), and CD19+ B cells (233.91 cells/µL), were lower than the normal range at admission. The lymphocyte count of CD16 + CD56+ NK cells of P2 was 10.91 cells/µL at admission, which was lower than the normal range. The lymphocyte counts of P3 were approximately normal. In addition, whole-genome sequencing revealed a novel missense mutation in signal transducer and activator of transcription 3 (*STAT3*) in P1. The whole-genome sequencing of P2 also revealed *STAT3* mutations, while that of P3 revealed caspase-recruitment domain 9 (*CARD9*) mutations.

**Table 1:** Clinical characteristics of the three patients with *T. marneffei* infections.

Patient	Sex	Age	Clinical manifestations	Complication	Mutant gene	Confirmed pathogenic specimens
P1	Male	13 years	Cough, dyspnea, malnutrition, a skin lesion, lymphadenectasis, and hoarseness	ARDS	STAT3,c.A1593T, p.K531	*Talaromyces marneffei, Candida albicans*
P2	Male	1 year	Cough, dyspnea, and hoarseness	ARDS	STAT3,c.1679_1681delCCT,p.Ser560del	*Talaromyces marneffei*,
P3	Female	1 year	Fever, loss of weight, cough, diarrhea, lymphadenectasis, and hoarseness	Sepsis	CARD9,c.1118G>C,p.R373P	*Talaromyces marneffei*

ARDS, acute respiratory distress syndrome.

**Table 2:** Laboratory findings of the three patients with *T. marneffei* infections.

Laboratory marker	P1	P2	P3
WBC (×109/L)	5.9 (5.0–12.0)	18.4 (5.0–12.0)	9.4 (5.1–14.1)
ANC (×109/L)	4.9 (2–7.2)	7.54 (2.0–7.2)	4.95 (0.8–5.8)
Hb (g/L)	99 (105–145)	124 (105–145)	94 (107–141)
PLT (×109/L)	460 (140–440)	497 (140–440)	497 (190–524)
CRP (mg/L)	14.25(<8.2)	N/A	27.28 (0–6)
PCT (ng/mL)	0.18 (<0.1)	N/A	0.02 (<0.5)
IgG (g/L)	17.1 (6.36–13.24)	12.9 (3.82–10.58)	42.4 (3.82–10.58)
IgA (g/L)	1.07 (0.49–2.29)	0.24 (0.14–1.14)	0.42 (0.14–1.14)
IgM (g/L)	0.61 (0.42–1.46)	2.62 (0.4–1.28)	1.86 (0.4–1.28)
IgE (IU/mL)	5,310 (0–200)	422 (0–60)	46 (0–60)
C3 (g/L)	1.02 (0.85–1.6)	0.93 (0.8–1.5)	0.68 (0.8–1.5)
C4 (g/L)	0.3 (0.14–0.43)	0.13 (0.12–0.4)	0.17 (0.12–0.4)
CD3 + CD4+ (Th cells) (cells/µL)	323.88 (345–2,350)	664.87 (410–1,590)	703.83 (410–1,590)
CD3 + CD8+ (Ts cells) (cells/µL)	576.34 (314–2,080)	418.42 (190–1,140)	250.16 (190–1,140)
CD19+ (B cells) (cells/µL)	233.91 (240–1,317)	798.31 (90–660)	810.15 (90–660)
Th/Ts (%)	0.56 (0.47–2.05)	1.59 (0.68–2.47)	2.81 (0.68–2.47)
CD16 + CD56 + (NK cells) (cells/µL)	75.91 (210–1,514)	10.91 (90–590)	187.45 (90–590)

WBC, white blood count; ANC, absolute neutrophil count; Hb, hemoglobin; PLT, platelets; CRP, C-reactive protein; Th, helper T; Ts, suppressor T; NK, natural killer.

### 3.2 Diagnostic assessment

**Figure 1:**
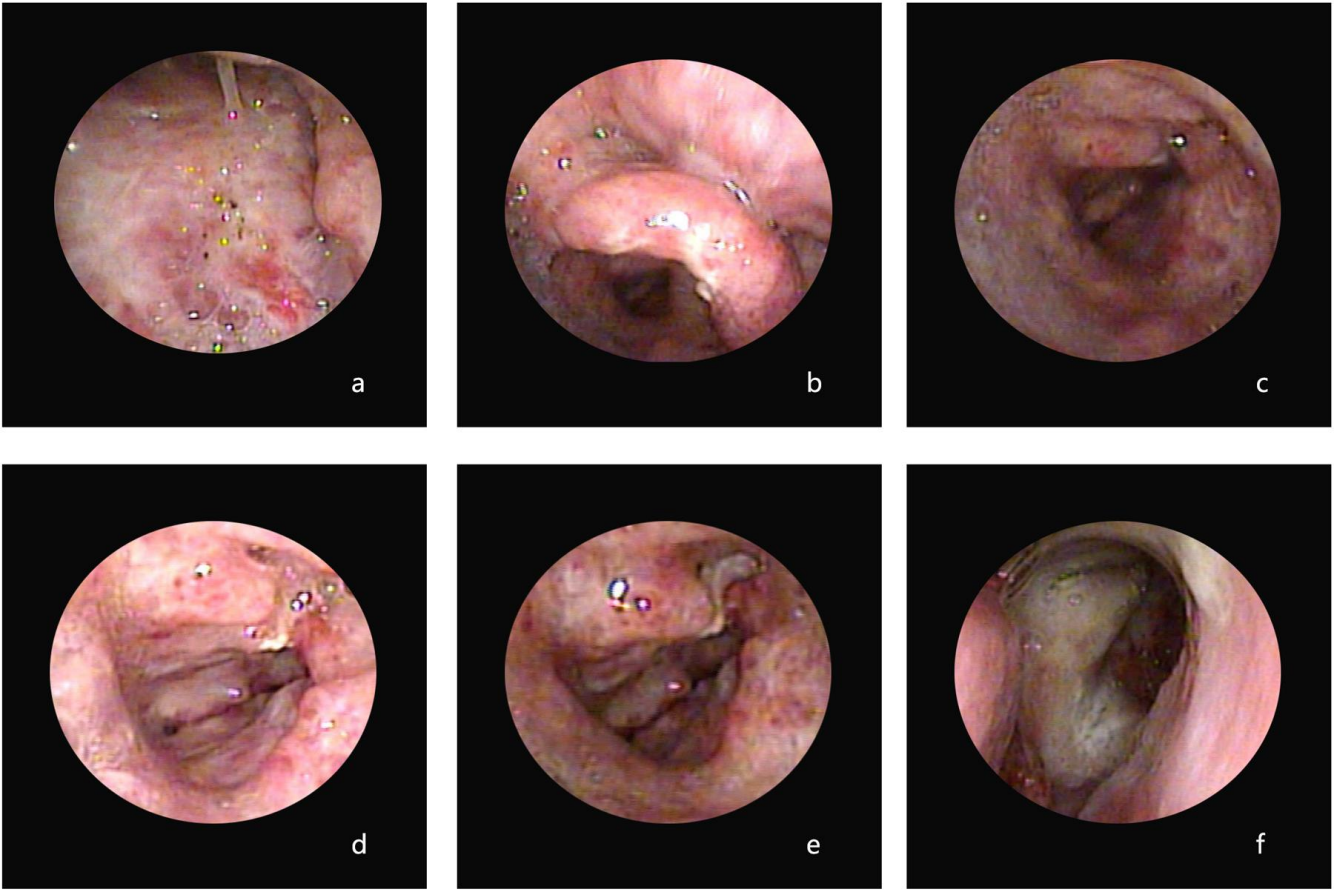
Images from the nasopharyngolaryngoscopy of P1 illustrating diffuse inflammation in the bilateral nasal cavity, posterior pharyngeal wall, and laryngeal mucosa, accompanied by a significant presence of purulent secretions on the surface. Additionally, vocal cord edema, unclear structure, and narrowing of the glottic area were observed **(a–f)**.

**Figure 2:**
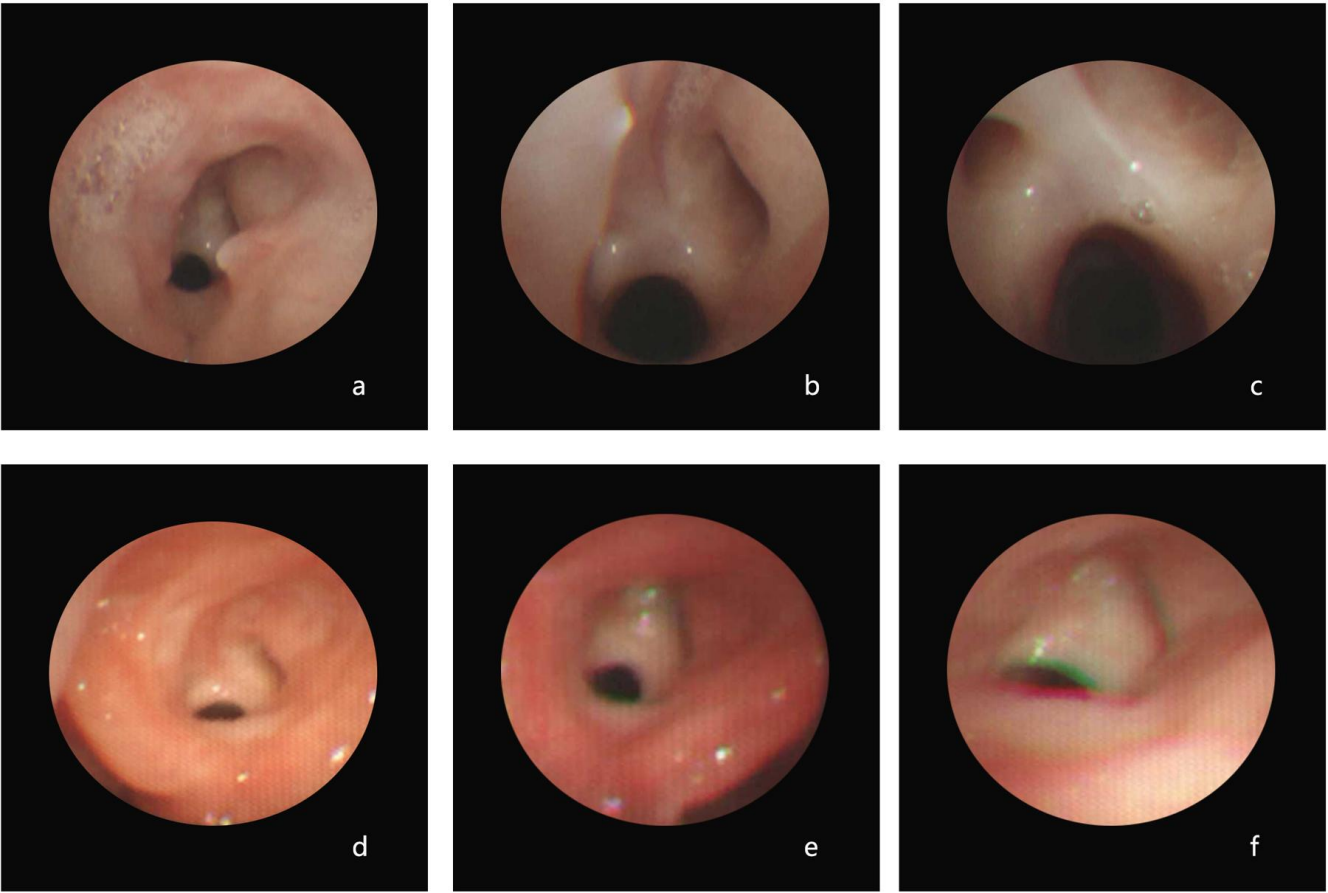
Electronic bronchoscopy of P2 revealed the following: in 2019 **(a–c)**, there was an abnormal glottic structure with hyperplasia of the granulation tissue and inflammatory stenosis of the subglottic airway; in 2020 **(d)**, the abnormal glottic structure accompanied by an obstruction due to hyperplasia of subglottic granulation tissue; and in 2021 **(e,f)**, the presence of a laryngeal web was observed.

**Figure 3:**
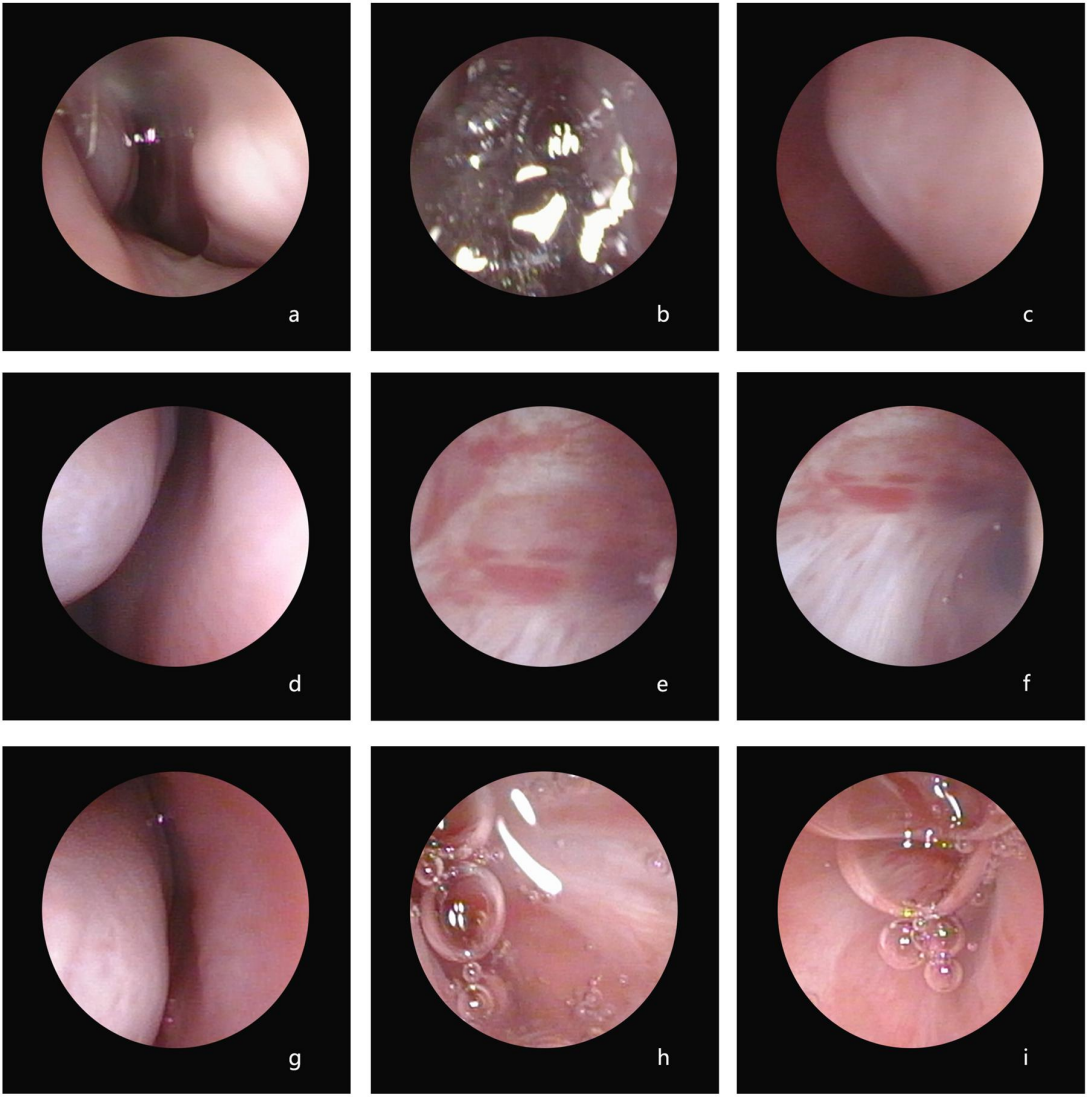
Nasopharyngolaryngoscopy in P3. Preoperative findings revealed posterior nostril atresia **(a–c)**. The postoperative assessment following posterior rhinoplasty demonstrated hyperplasia of nasopharyngeal scar tissue **(d–f)**. Additional observations at follow-up included rhinitis, nasopharyngeal adhesions, and posterior nostril atresia **(g–i)**.

All the children showed evidence of pharyngeal and laryngeal injuries. P1 exhibited pharyngeal and laryngeal injuries when he was admitted in 2015, and the nasopharyngolaryngoscopy results revealed diffuse inflammation in the bilateral nasal cavity, posterior pharyngeal wall, and laryngeal mucosa, accompanied by a significant presence of purulent secretions on the surface. In addition, vocal cord edema, unclear structure, and narrowing of the glottic area were observed (Figures 1a–f). P2 had pharyngeal and laryngeal injuries at admission and follow-up. Electronic bronchoscopy in 2019 revealed an abnormal glottic structure with hyperplasia of granulation tissue and inflammatory stenosis of the subglottic airway (Figures 2a–c). A follow-up was conducted for this child in 2020, and bronchoscopy revealed abnormal glottic structure accompanied by an obstruction due to hyperplasia of subglottic granulation tissue (Figure 2d). Finally, a laryngeal web was found in 2021 (Figures 2e–f). P3 had pharyngeal ulcers at admission in November 2021. Subsequently, posterior nostril atresia was diagnosed via nasopharyngolaryngoscopy conducted in February 2022 (Figures 3a–c). Therefore, the patient underwent posterior rhinoplasty surgery. After surgery, nasopharyngolaryngoscopy in P3 revealed significant hyperplasia of nasopharyngeal scar tissue (Figures 3d–f). During the follow-up in 2023, nasopharyngolaryngoscopy revealed rhinitis, nasopharyngeal adhesions, and posterior nostril atresia (Figures 3g–i). Sputum cultures indicated *T. marneffei* in all three patients.

### 3.3 Therapeutic intervention

In P1, mechanical ventilation was needed on two occasions because of the complications of acute respiratory distress syndrome and upper airway obstruction due to granulation tissue. We administered amphotericin B, voriconazole, and imipenem-cilastatin sodium intravenously for 2 weeks and administered itraconazole orally for 2 months as antifungal agents. P2 required mechanical ventilation because of the complications of subglottic stenosis and acute respiratory failure. We administered voriconazole and itraconazole as antifungal agents. P3 required amphotericin B and itraconazole as antifungal agents.

### 3.4 Follow-up and outcomes

P1 showed a good clinical response. P2 showed good clinical results and was discharged from the hospital. After discharge, P2 underwent bronchoscopy, and the glottic injury remained. P3 showed good clinical results and was discharged from the hospital. During the follow-up period, adhesion of the nasopharynx was observed in P3 and was treated with surgery. At the time of publication, P3 had exhibited no cough or wheezing but was still breathing with an open mouth.

## 4 Discussion and conclusion

*T. marneffei* infection frequently presents with characteristic clinical features such as fever, peripheral lymphadenectasis, respiratory symptoms, weight loss, skin lesions, and gastrointestinal symptoms (7). Pharyngeal and laryngeal injuries caused by *T. marneffei* infection have rarely been reported in children. We present three cases of pharyngeal and laryngeal injuries from *T. marneffei* infection in HIV-negative children. The clinical manifestations observed in these pediatric patients with *T. marneffei* infection were not typical. However, respiratory symptoms caused by *T. marneffei* infection, including cough, wheezing, and hoarseness, are the earliest clinical manifestations (8). Our patients presented with pharyngeal and laryngeal injuries and were diagnosed by electronic nasopharyngolaryngoscopy and bronchoscopy. Although the clinical histories of these patients were different, the results of electronic nasopharyngolaryngoscopy and bronchoscopy indicated varying degrees of injury to the pharynx. Patient 3 was the most severely injured child, and she underwent nasopharyngeal surgery. In a previous study, fever and cough were found to be the main manifestations of a *T. marneffei* infection (9). Furthermore, the extrapulmonary organs targeted by a *T. marneffei* infection included bone, skin, lymph nodes, and/or the central nervous system (10). Although our patients were HIV-negative, they had serious pharyngeal and laryngeal injuries. *T. marneffei* primarily spreads through the respiratory tract and compromised skin, but the potential mechanisms of susceptibility to the fungus remain incompletely understood (11). Pharyngeal and laryngeal injuries are very rare, and the detailed mechanisms involved are unclear. Nevertheless, clinicians are advised to implement more aggressive treatment strategies for a *T. marneffei* infection when pharyngeal and laryngeal injuries are detected. A rapid and accurate diagnosis is crucial for improving the prognosis. Therefore, in this study, the clinical manifestations of *T. marneffei* infections with pharyngeal and laryngeal injuries in HIV-negative children were reviewed and analyzed.

In the children with *T. marneffei* infections included in this study, pharyngeal and laryngeal injuries were the most important symptoms. There is currently no standard treatment for a *T. marneffei* infection with pharyngeal and laryngeal injuries. When patients are diagnosed, effective antifungal treatments and supportive therapy are essential (12). Among our three patients, P1 and P2 were mainly treated with antifungal therapy. However, their glottic structures failed to recover completely after treatment. In P3, the surgical procedure helped the patient recover her glottic structure. Future investigations should explore therapies for patients with a *T. marneffei* infection. An effective and appropriate treatment for pharyngeal and laryngeal injuries still requires further investigation.

The susceptibility of patients with certain types of IEIs to fungal infection is particularly pronounced. The prevalence of children being diagnosed with an IEI and a *T. marneffei* infection has increased in recent years. Some genetic mutations, including mutations in signal transducer and activator of transcription 1 (*STAT1*), *STAT3*, *CARD9,* and so on, have been reported to be associated with a *T. marneffei* infection (13–15). The gene profiles of patients with IEIs who are susceptible to a *T. marneffei* infection suggest the potential involvement of cellular immunity (16). In our study, two patients were confirmed to have *STAT3* gene mutations and the other had *CARD9* gene mutations. Whether genetic mutations are involved in pharyngeal and laryngeal injuries is unclear. In the future, more cases should be collected for a mechanistic investigation.

With respect to treatment, global guidelines suggest that antifungal therapy should be promptly administered to patients with a *T. marneffei* infection, especially in immunocompromised patients. Antifungal therapy involves induction, consolidation, and maintenance phases (17). However, there is currently no standard treatment for a *T. marneffei* infection in HIV-negative pediatric patients. However, induction therapy with amphotericin B for more than 7 days has a good prognosis in these patients (2), and long-term consolidation and maintenance therapy with itraconazole is recommended after induction therapy (18).

In conclusion, damage to the pharyngeal and laryngeal compartments appears to be an important clinical manifestation in HIV-negative pediatric patients with a *T. marneffei* infection. Further studies are required to develop therapies for pharyngeal and laryngeal injuries in patients with *T. marneffei* infections.

## Data Availability

The original contributions presented in the study are included in the article/Supplementary Material, further inquiries can be directed to the corresponding author.
